# A Mixed-chimerism Protocol Utilizing Thymoglobulin and Belatacept Did Not Induce Lung Allograft Tolerance, Despite Previous Success in Renal Allotransplantation

**DOI:** 10.1097/TXD.0000000000001150

**Published:** 2021-05-25

**Authors:** Wiebke Sommer, Jane M. O, Kurt B. Pruner, Abbas Dehnadi, Kyu Ha Huh, Kortney A. Robinson, Isabel Hanekamp, Ivy Rosales, Alison S. Bean, Josh Paster, Tetsu Oura, Rex Neal Smith, Robert Colvin, Gilles Benichou, Tatsuo Kawai, Joren C. Madsen, James S. Allan

**Affiliations:** 1 Center for Transplantation Science, Massachusetts General Hospital and Harvard Medical School, Boston, MA.; 2 Department of Cardiac Surgery, University of Heidelberg, Heidelberg, Germany.; 3 Department of Transplantation Surgery, Severance Hospital, Yonsei University College of Medicine, Seoul, Korea.; 4 Department of Surgery, Beth Israel Deaconess Medical Center, Boston, MA.; 5 Department of Pathology, Massachusetts General Hospital and Harvard Medical School, Boston, MA.; 6 Division of Cardiac Surgery, Massachusetts General Hospital, Boston, MA.; 7 Division of Thoracic Surgery, Massachusetts General Hospital, Boston, MA.

## Abstract

**Background.:**

In kidney transplantation, long-term allograft acceptance in cynomolgus macaques was achieved using a mixed-chimerism protocol based on the clinically available reagents, rabbit anti-thymocyte globulin (ATG), and belatacept. Here, we have tested the same protocol in cynomolgus macaques transplanted with fully allogeneic lung grafts.

**Methods.:**

Five cynomolgus macaques underwent left orthotopic lung transplantation. Initial immunosuppression included equine ATG and anti-IL6RmAb induction, followed by triple-drug immunosuppression for 4 mo. Post-transplant, a nonmyeloablative conditioning regimen was applied, including total body and thymic irradiation. Rabbit ATG, belatacept, anti-IL6RmAb, and donor bone marrow transplantation (DBMT) were given, in addition to a 28-d course of cyclosporine. All immunosuppressant drugs were stopped on day 29 after DBMT.

**Results.:**

One monkey rejected its lung before DBMT due to AMR, after developing donor-specific antibodies. Two monkeys developed fatal post-transplant lymphoproliferative disorder, and both monkeys had signs of cellular rejection in their allografts upon autopsy. The remaining 2 monkeys showed severe cellular rejection on days 42 and 70 post-DBMT. Cytokine analysis suggested higher levels of pro-inflammatory markers in the lung transplant cohort, as compared to kidney recipients.

**Conclusion.:**

Although the clinically applicable protocol showed success in kidney transplantation, the study did not show long-term survival in a lung transplant model, highlighting the organ-specific differences in tolerance induction.

## Abstract 2

Supplemental Digital Content is available in the text.

## INTRODUCTION

Tolerance induction in solid-organ transplantation remains the ideal treatment for transplant recipients, avoiding side-effects of long-term immunosuppression, as well as the risk of graft loss due to acute or chronic rejection. Several groups have shown, in both preclinical and clinical studies, that protocols based on mixed-chimerism have the potential to lead to long-term allograft acceptance without maintenance immunosuppression. However, the majority of studies have been conducted in kidney transplantation models.

In lung transplantation, we have previously shown that long-term allograft acceptance in nonhuman primates (NHPs) can be achieved with a delayed mixed hematopoietic chimerism approach by using equine anti-thymocyte globulin (ATG), anti-CD8 mAb, anti-CD154 mAb, and IL-6R blockade. Three out of 4 animals accepted their allografts long-term without evidence for rejection. Similar results were seen with kidney transplantation and heart/kidney transplantation in NHPs. However, anti-CD8 and anti-CD154 mAbs are not available for clinical use.

Our group recently reported long-term allograft acceptance in kidney transplanted NHPs using a protocol that utilized thymoglobulin and belatacept, reagents already in clinical use.^1^ We, therefore, aimed to adapt this kidney protocol to generate a tolerance protocol applicable to human lung transplantation.

## MATERIAL AND METHODS

### Animals

Lung transplantations were performed using Mauritius cynomolgus macaques weighing 4–8 kg (Charles River Primates, Wilmington, MA). Recipient and donors were ABO-matched and major histocompatibilty complex (MHC) genotyped for cynomolgus leukocyte MHC genes as previously described^2^ (Figure S1, **SDC**, http://links.lww.com/TXD/A325). All procedures were performed in accordance with the National Institute of Health “Guidelines for the Care and Use of Laboratory Animals” and were approved by the Massachusetts General Hospital Institutional Animal Care and Use Committee. Mauritius cynomolgus macaques were also used for kidney transplantation, as previously reported.^1^

### Treatment Regimen

**FIGURE 1.**
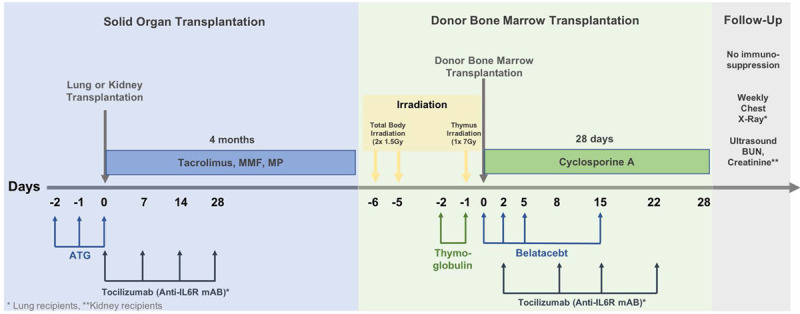
Schematic for protocol of delayed tolerance induction following organ transplantation. Animals received equine ATG for induction therapy before transplantation and were kept on triple-drug immunosuppression for 4 mo. Thereafter, a nonmyeloablative conditioning regimen including total body irradiation (TBI), thymic irradiation (TI), and thymoglobulin (rabbit ATG) was performed. After donor bone marrow transplantation, belatacept was administered as well as a 28-d course of cyclosporine. Lung animals received an anti-IL6 receptor blocker following initial organ transplantation and DBMT. All immunosuppressive medication was stopped thereafter (kidney protocol redrawn from Hotta et al)^1^. ATG, anti-thymocyte globulin; DBMT, donor bone marrow transplantation.

Animals undergoing left-sided lung transplantation received equine ATG for lymphocyte depletion before lung transplantation (50 mg/kg, days 2, 1, and 0, ATGam, Pharmacia and Upjohn, Kalamazoo, MI) as well as anti-IL6 receptor blocker (10 mg/kg, days 0, 7, 14, 28; Tocilizumab, Genentech, San Francisco, CA) for reduction of perioperative and postoperative inflammatory responses. Recipients were then maintained on triple-drug immunosuppression, similar to kidney recipient animals. Tacrolimus (target trough level 20–30 ng/dL, Astellas Pharma Inc., Osaka, Japan), as well as steroids, were given daily by intramuscular injections. Mycophenolate mofetil (200 mg/day, Roche Inc., Nutley, NJ) was administered orally. After 4 mo, animals underwent nonmyeloablative conditioning including total body irradiation, thymic irradiation, and lymphocyte depletion using rabbit ATG. In addition, lung recipients received 4 doses of anti-IL6R on days 0, 7, 14, and 28 (Figure 1). Donor bone marrow transplantation (DBMT) in both groups was performed using cryopreserved donor bone marrow as described previously.^3,4^ Immunosuppressive drugs were stopped after a 28-day course of cyclosporine A (target trough level 200–300 ng/dL).

Animals undergoing kidney transplantation were treated similarly as previously reported (Figure 1, redrawn from Hotta et al).^1^

### Flow Cytometry Analysis

Single-cell suspensions were prepared from peripheral blood as previously described and stained with fluorochrome-conjugated antibodies specific toward CD3ε (SP34), CD4 (L200), CD8 (SK1), CD21 (B-ly4), CD27 (M-T271), CD20 (2H7), CD16 (NKp16), CD25 (2A3), IgG3k (all from BD Pharmingen, San Jose, CA), CD28 (CD28.2), CD95 (DX2), CD16 (3G8), CD25 (BC96) (all from Biolegend, San Diego, CA), and CD159a (NKG2A) (Beckman Coulter, Brea, CA). For intracellular FoxP3 expression, cells were permeabilized (eBioscience, San Diego, CA) and stained using an anti-FoxP3 mAb (236A\E7, Invitrogen, Carlsbad, CA).

For chimerism detection, donors were selected for specific expression of class I MHC antigen H38; the recipients did not express this antigen. Chimerism analysis was done by flow cytometry using a specific anti-MHC mAb (H38, One Lambda, Inc., Canoga Park, CA).

Flow cytometry was performed using a FACSverse (BD Biosciences, San Jose, CA) or Accuri Flow Cytometer (BD Biosciences, San Jose, CA) and analyzed using FlowJo software (Flowjo LLC, Ashland, OR).

### Alloantibody Analysis

Donor-specific alloantibodies directed towards T cells and B cells were detected using flow cytometry as previously described.^5^ Results are reported as “fold change” of mean fluorescence intensity, as compared with serum obtained before transplantation from the respective animal.

### Alloresponse Analysis by ELISpot

Cryopreserved recipient, donor, and third-party peripheral blood mononuclear cells (PBMCs) were thawed and rested overnight in complete R10 media. ELISpot plates (Millipore, Bedford, MA) precoated with IFN-g (Mabtech, Nacka Strand, Sweden) were blocked for 30 min using serum-containing media. Responder cells as well as irradiated stimulator cells were plated (150.000/well) and incubated at 37°C. Media and autologous cells were used as negative control, phytohemagglutinin (Sigma-Aldrich, St. Louis, MO) was used as a positive control. After 48 h, plates were washed, and a biotinylated anti-IFN-γ detection antibody (Mabtech, Nacka Strand, Sweden) was added. After 1 h, streptavidin horseradish peroxidase conjugate (Dako, Glostrup, Denmark) was added. Finally, TMB substrate (Mabtech, Nacka Strand, Sweden) was added for plate development, and the reaction was stopped using deionized water. Plates were read using an ELISpot image analyzer (CTL Inc., Cleveland, OH).

### Mixed Lymphocyte Reaction

Cryopreserved recipient and third-party PBMCs were thawed and rested overnight in complete R10 media. Responder cells were then labeled with carboxyfluorescein succinimidyl ester (CFSE) (Life Technologies, Waltham, MA) at a concentration of 3 μM per 10^7^ cells at 37°C for 8 min and cultured in 96-well flat-bottom plates with irradiated donor and third-party PBMC (each 400.000/well). After 5 d, the cells were stained with antibodies, and CFSE dilution was assessed by flow cytometry.

### Cytokine Analysis

Serum from kidney and lung transplant animals was analyzed 30 d before BMT (pre-BMT) and compared with similar serum samples from 30 d after BMT (post-BMT). Cytokines were measured using a magnetic bead-based Luminex multiplex cytokine kit (Millipore, Billerica, MA). Briefly, recipient serum was incubated overnight at 4°C with magnetic beads, specifically coated with antibodies for IL-1β, IL-6, IL-1RA, IFN-γ, IL-10, IL-18, and MIP-1α. A biotinylated detection antibody was then added and incubated for 1 h at room temperature. Following this, streptavidin–phycoerythrin was added for 30 min. After this, samples were washed and analyzed using Luminex MAGPIX as well as xPONENT software.

### Graft Monitoring and Histologic Analysis

Lung recipients underwent weekly chest radiographies as well as open lung biopsies. One biopsy was performed routinely before bone marrow transplantation to rule out evidence for rejection during triple-drug immunosuppression. All samples as well as tissue obtained during the autopsy were formalin-preserved and underwent routine hematoxylin and eosin staining. For detection of C4d deposition, paraffin-fixed sections were stained using a polyclonal anti-C4d (Biomedica, Vienna, Austria). Histologic analysis was performed by transplant pathologists blinded to the protocol (I.R., R.N.S., and R.B.C.). Rejection was scored following the revised International Society for Heart and Lung Transplantation grading system for acute cellular rejection.^6^

Kidney grafts were monitored by serum creatinine level analysis as well as serial biopsies as previously published.^1^

### Statistical Analysis

Data are given as mean ± SD unless otherwise stated. Allograft survival was analyzed using the Kaplan–Meier method. Values <0.05 were considered statistically significant.

## RESULTS

### Lung Recipient Failed to Develop Allograft Acceptance

**FIGURE 2.**
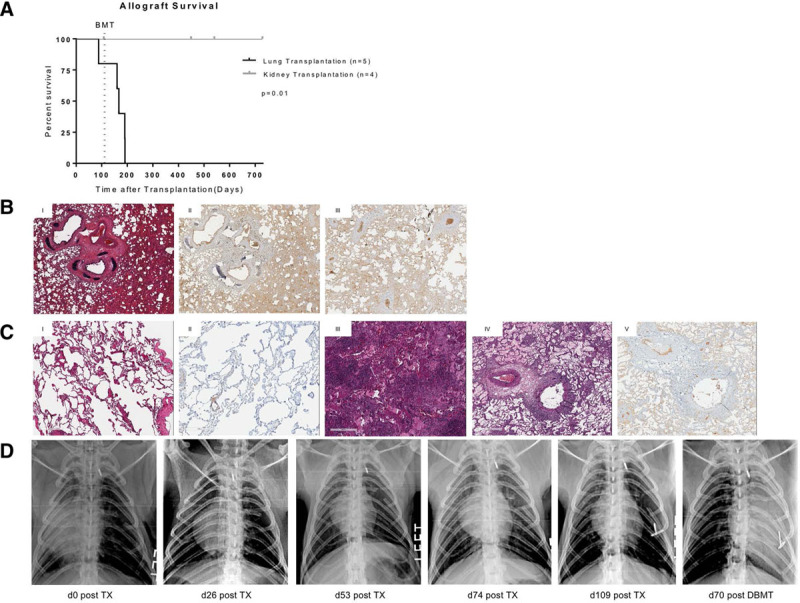
A, Kaplan–Meier analysis of graft survival following lung (black curve) and kidney (gray curve) transplantation. Allograft survival following kidney transplantation was significantly superior as compared to animals undergoing lung transplantation (*P* = 0.01). B, Pathology of lung allografts. (1) Hematoxylin and eosin staining of an allograft lung at the time of euthanasia (M8216) on day 78 after lung transplantation before DBMT. The histology shows signs of the severe antibody as well as mild acute cellular rejection (ISHLT grading: A1, B1R, AMR, C4d pos) with thickening of alveolars as well as lymphocyte infiltration. (II) and (III): Histology of the allograft lung of M8216 at time of euthanasia showing positive C4d staining, suggesting severe antibody-mediated rejection. C, Pathology of lung allografts sampled pre-DBMT (I and II) of M7416 showing no signs of cellular or antibody-mediated rejection. Upon euthanasia on day 70 post-DBMT III–V), histology showed severe acute cellular as well as antibody-mediated rejection with positive C4d staining (ISHLT grading: A4, B2R, AMR, C4d pos). D, Chest radiographs of M7416 of the postoperative course showing a well-aerated left allograft lung pre-DBMT, but diffuse infiltration after DBMT on day 70 after BMT indicating graft rejection. DBMT, donor bone marrow transplantation; ISHLT, International Society for Heart and Lung Transplantation.

Animals undergoing life-sustaining kidney transplantation developed long-term graft acceptance as previously published, surviving until days 449, 540, and 728 after bone marrow transplantation, respectively^1^ (Figure 2A, Figure S1B, **SDC**, http://links.lww.com/TXD/A325). In contrast, recipients of left-sided lung transplants did not develop long-term allograft acceptance. One monkey (M8216) showed respiratory distress on day 78 after transplantation within the period of triple-drug immunosuppression and was diagnosed with graft failure due to antibody-mediated rejection on autopsy. Histology revealed endarteritis as well as interstitial capillaritis and positive C4d staining (Figure 2B). A fatal post-transplant lymphoproliferative disorder (PTLD) was diagnosed in two other animals (M6516 and M5816) on days 41 and day 72 after DBMT; however, on autopsy, there were also signs of acute cellular rejection in the allograft lungs of both animals. Two animals (M7416 and M4116) showed severe acute cellular rejection on days 42 and 70 after DBMT, without showing signs of rejection in the allograft biopsy performed before bone marrow transplantation (Figure 2C and D). One of these monkeys, M7416 also developed donor-specific anti-T-cell and anti-B-cell antibodies, leading to humoral rejection in addition to acute cellular rejection.

### Cellular Reconstitution of Lung and Kidney Recipients Showed Dominance of CD8^+^ Lymphocytes

**FIGURE 3.**
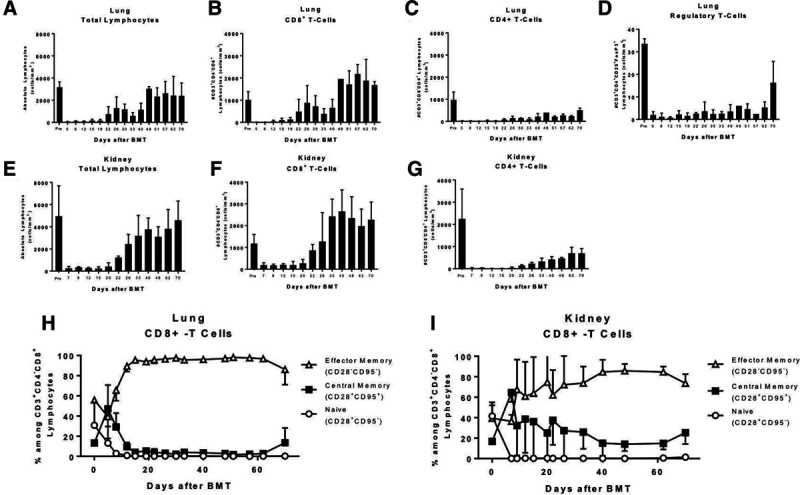
Cellular lymphocyte reconstitution in allograft recipients. A, Lung recipients showed effective depletion of lymphocytes during conditioning and DBMT; reconstitution of lymphocytes was similar between lung and kidney allograft recipients (E). Both groups showed an overspilling reconstitution of CD3+CD8+ lymphocytes, with overall numbers being higher in the kidney group (B, F). Similarly, CD3+CD4+ lymphocytes underwent effective depletion, the kidney group showing higher absolute numbers upon reconstitution as compared with lung recipients (C, G). Lung recipients failed to reconstitute regulatory T cells to pre-DBMT levels throughout the entire postoperative monitoring phase (D). Further differentiation of CD8+ T-lymphocytes revealed a high percentage of effector memory T cells in both groups upon reconstitution. In addition, kidney recipients expressed a larger proportion of central memory T cells, whereas lung animals failed to reconstitute central memory cells (H, I). DBMT, donor bone marrow transplantation.

Lung recipients undergoing conditioning for DBMT showed effective depletion of the lymphocyte compartment including CD4+ and CD8+ T cells, starting on the day of DBMT until day 19 after BMT (Figure 3A–D). CD3+CD4+ cells failed to recover to pre-transplant numbers and remained low until allograft failure. In contrast, CD3+CD8+ T cells show an exuberant recovery, with post-transplant numbers higher than pre-transplant values, until the allografts were rejected (Figure 3B). Similar depletion and reconstitution were seen in the kidney group; however, the absolute number of CD4+ cells was higher in the kidney group, as compared with the lung group (Figure 3E–G). Upon reconstitution, the majority of CD8+ cells were characterized as CD3+CD8+CD28−CD95− effector memory cells, whereas central memory cells (CD3+CD8+CD28+CD95+ cells) and naive CD8+ cells (CD3+CD8+CD28+CD95− cells) remained low after DBMT in the lung group (Figure 3H). However, animals that underwent kidney transplantation showed a lower percentage of CD8+ effector memory cells and a higher percentage of central memory cells upon reconstitution, as compared with lung monkeys (Figure 3H and I). Regulatory T cells (CD3+CD4+CD25+FoxP3+) were depleted in the early phase after DBMT and remained markedly low, as compared to pre-transplant values until all allografts were rejected (Figure 3G). Data for T regulatory cells in the kidney group are not available.

### Lung Recipients Showed Similar Chimerism as Kidney Recipients

Following DBMT, lung recipients developed similar percentages of donor-derived chimerism as compared with kidney recipients. Interestingly, the 2 animals in the lung group that later developed PTLD (M6516 and M5816) showed higher lymphoid chimerism, as compared with the other 3 lung recipients, and both were still chimeric in all myeloid cell lines at the time of euthanasia, despite showing histologic signs of cellular rejection in the allograft (Figure S2A–C, **SDC**, http://links.lww.com/TXD/A325). All lung recipients showed expected levels of monocyte chimerism but slightly lower granulocyte chimerism, as previously seen in long-term tolerant lung recipients.^7^ No significant difference was detectable when comparing chimerism of lung and kidney recipients, and no durable chimeric state was seen in either group (Figure S2B, C, **SDC**, http://links.lww.com/TXD/A325).

### Cytokine Profile Suggests State of Elevated Inflammation in Lung Recipients as Compared with Kidney Recipients

**FIGURE 4.**
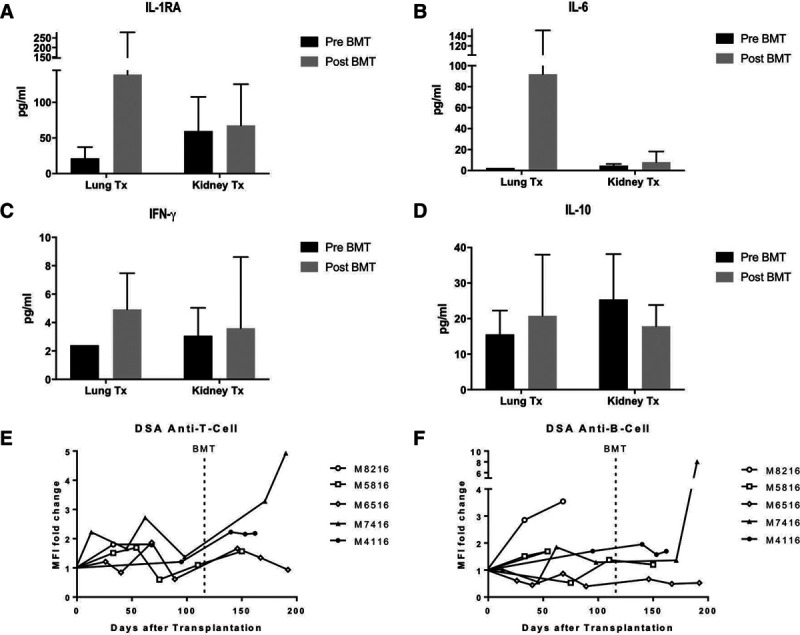
Serum cytokine levels before DBMT (day 30) and after DBMT (d30pBMT). Lung recipients showed higher levels of proinflammatory cytokines (IL-1RA, IL-6, and INF-g) after DBMT as compared with kidney recipients (A, B, C). Also, lung recipients had slightly higher levels of IL-10 after DBMT as compared with pre-DBMT, whereas kidney recipients showed lower levels of IL-10 after DBMT as compared with preBMT (D). (E, F) Donor-specific antibodies in lung transplant recipients. Animals showing signs of humoral rejection upon euthanasia also showed detectable anti-B-cell antibodies (M8216 and M7416) as well as anti-T-cell donor-specific antibodies (M7416 and M4116). DBMT, donor bone marrow transplantation.

Cytokine levels of kidney and lung transplant recipients showed differing results after bone marrow transplantation, when the induction of tolerance is expected to occur. Lung transplant recipient animals showed elevated levels of interferon-γ (4.9 ± 2.5 vs 3.6 ± 5.0 pg/mL) and IL-1RA (139.1 ± 140.8 vs 67.7.1 ± 57.8 pg/mL; *P* = 0.34) (Figure 4A and C). In lung transplant animals receiving anti-IL6 receptor antagonist after DBMT, serum IL-6 levels were higher as compared with kidney recipients (Pre-BMT: 2.4 vs 4.7 ± 1.7 pg/mL; *P* = 0.14; post-DBMT: 91.9 ± 59.9 vs 8.0 ± 10.2 pg/mL; *P* = 0.05) (Figure 4B). Serum levels of IL-10 were slightly higher in kidney recipients before DBMT, as compared with lung transplant recipients (25.4 ± 12.8 vs 15.6 ± 6.7 pg/mL; *P* = 0.26). However, post-DBMT levels were similar in both groups (17.9 ± 5.9 vs 20.8 ± 17.2 pg/mL; *P* = 0.66) (Figure 4D).

### Belatacept-based Regimen Led to High Incidence of PTLD in Nonhuman Primates After Lung Transplantation

Two lung recipient animals undergoing the belatacept-based regimen developed a PTLD. This incidence of PTLD is similar to that seen in other NHP lung protocols. One animal (M5816) still showed measurable serum levels of cyclosporine when pharyngeal tumors were seen on day 41 after DBMT. The other monkey (M6516) did not have detectable cyclosporine levels at the time of PTLD onset, on day 72 after DBMT.

### Development of Donor-specific Antibodies Led to Early Graft Failure in Lung Recipients but not Kidney Recipients

One lung recipient (M8216) developed anti-B-cell antibodies (3.5-fold increase in MFI compared with baseline MFI) in the period of triple-drug immunosuppression leading to antibody-mediated rejection and graft failure on day 78 post-transplantation (Figure 4F). A second lung recipient (M7416) developed high levels of anti-T-cell (4.9-fold increase in MFI) as well as anti-B-cell (8.03-fold increase in MFI) antibodies directed towards donor cells, contributing to a mixed picture of humoral and cellular rejection on day 70 post-DBMT. The remaining animals did not show antidonor antibody development (Figure 4E and F). In contrast, 3 kidney recipients developed permanent or transient donor-specific antibodies against MHC class II without functional allograft impairment.^2^

### Lung Recipient Animals Showed Elevated Cellular Anti-donor Response Upon Rejection

Cellular antidonor response was assessed in all lung transplant recipients. Both animals (M4116 and M7416) that rejected their allo-lung due to acute cellular rejection showed markedly increased antidonor CD8+ response at the time of euthanasia, as compared to pre-transplant. These results differ widely from kidney recipients, which showed a consistent decrease in antidonor CD8+ response following BMT, as compared with pre-DBMT.

Although lung recipients showed a similar increase in antidonor CD4^+^ response following DBMT, as did kidney recipients, lung recipients showed no rise in donor-reactive regulatory T cells following DBMT. In contrast, kidney recipients accepting their allograft showed a significant increase in FoxP3^+^ regulatory cell expansion in the post-DBMT period, as compared to before DBMT (Figure S3A–C, **SDC**, http://links.lww.com/TXD/A325).

Similar results were detectable in IFN-γ specific ELISpot assays. M4116 showed an increasing antidonor response during the delayed period of triple-drug immunosuppression and increased further after DBMT and time of euthanasia. Similarly, M7416 showed an initial decrease of antidonor immune response on day 63 after transplantation, but his antidonor response increased thereafter, peaking at the time of euthanasia (Figure S3D, **SDC**, http://links.lww.com/TXD/A325).

## DISCUSSION

The data reported here show that durable allograft acceptance was not achieved in lung transplant recipient animals undergoing a similar protocol that was able to induce long-term allograft acceptance in kidney transplantation, suggesting the need for organ-specific tolerance induction protocols. Solid organs are known to have differing thresholds for allograft acceptance, with kidney and liver grafts being more prone to tolerance than thoracic organs.^8-14^

Following bone marrow transplantation, lung transplant recipient animals showed a slow recovery of the CD3+CD4+ compartment of lymphocytes; however, none of the animals recovered to the pre-transplant state. In contrast, CD3+CD8+ lymphocytes showed an exuberant reconstitution with 1.5-fold as many CD8+ lymphocytes, when compared with pre-transplantation. The vast majority of these were effector memory T cells. Also, the ratio of CD8+effector to CD8+ central memory T cells differed between accepting kidney animals and rejecting lung animals, with a higher percentage of central memory T cells and a lower percentage of effector memory T cells in the kidney group. In combination with the failed reconstitution of regulatory T cells after bone marrow transplantation, the cellular milieu in the lung transplant cohort suggests elevated antidonor reactivity with a lack of pro-regulatory mechanisms.

The importance of FoxP3+ regulatory T cells for the development of durable allograft acceptance has been described by several groups in the past. Duran-Struuck and colleagues were able to achieve long-term kidney acceptance in a mixed-chimerism model with delayed organ transplant, showing that animals receiving ex vivo expanded Tregs developed prolonged allograft acceptance. Animals not receiving expanded Tregs failed to become tolerant.^15^ An analysis of human lung transplant recipients showed a positive correlation between higher numbers of Tregs in peripheral blood early after transplant surgery with a lower incidence of chronic rejection.^16^

Although 2 monkeys died for malignancies while still being chimeric in the early phase after BMT, none of the remaining lung transplant recipients developed durable chimerism. Similarly, all 3 monkeys showing long-term allo-kidney acceptance only developed transient chimerism. In a previously published protocol in NHPs in which 3 animals developed long-term lung allograft acceptance, 2 tolerant animals showed durable donor chimerism.^7^ Therefore, the discussion of whether durable or transient chimerism is required for organ acceptance might also be looked at from an organ-specific perspective. With regard to the kidney, durable chimerism does not appear to be necessary for long-term allograft acceptance; however, durable chimerism in lung transplantation appears to be beneficial.

Data from this protocol showed higher serum levels of pro-inflammatory cytokines in lung recipients, as compared with kidney recipients immediately following DBMT, suggesting that a higher state of inflammation prevented tolerance induction in lung transplant recipients. This is not surprising given the lungs’ role as a barrier organ. Because lung recipients showed no clinical signs of infection before conditioning and DBMT, elevated inflammatory cytokines may be explained by activation of antidonor adaptive immunity. Several clinical studies in human kidney recipients showed that an elevated IFNγ-mediated immune response immediately after transplantation is associated with an increased risk for acute and chronic rejection. This may be explained by the presence of a larger number of IFN-γ producing alloreactive memory T cells.^17-19^ Also, IFN-γ-induced chemokines are known to be elevated in transplant recipients with signs of rejection as compared to recipients with normal allograft function.^20^ Similarly, IL-1RA, which was significantly higher in lung animals as compared with kidney animals, is known to increase as a response to noninfectious inflammatory stimuli^21^ and may therefore add to the overall elevated state of inflammation in the lung recipients in our study.

In conclusion, our clinically applicable thymoglobulin and belatacept-based protocol did not induce long-term tolerance in this model of NHP lung transplantation, suggesting the existence of organ-specific differences in the ability to achieve immunological tolerance. For tolerance induction in lung transplantation, approaches that include greater regulatory T-cell induction while minimizing overall immunosuppression and inflammation are necessary.

## ACKNOWLEDGMENTS

Antibodies used in these studies were produced by the NIH Nonhuman Primate Reagent Resource funded by NIH grant OD010976 and NIAID contract HHSN272200130031C.

## Supplementary Material


