# Supplementary material for: A Mixed-chimerism Protocol Utilizing Thymoglobulin and Belatacept Did Not Induce Lung Allograft Tolerance, Despite Previous Success in Renal Allotransplantation
Source: Transplant Direct. 2021 May 25;7(6):e705. doi: 10.1097/TXD.0000000000001150 (PMC8154498; doi:10.1097/TXD.0000000000001150)
Supplement: Supplementary file 1 [file txd-7-e705-s001.pdf]

S1

A

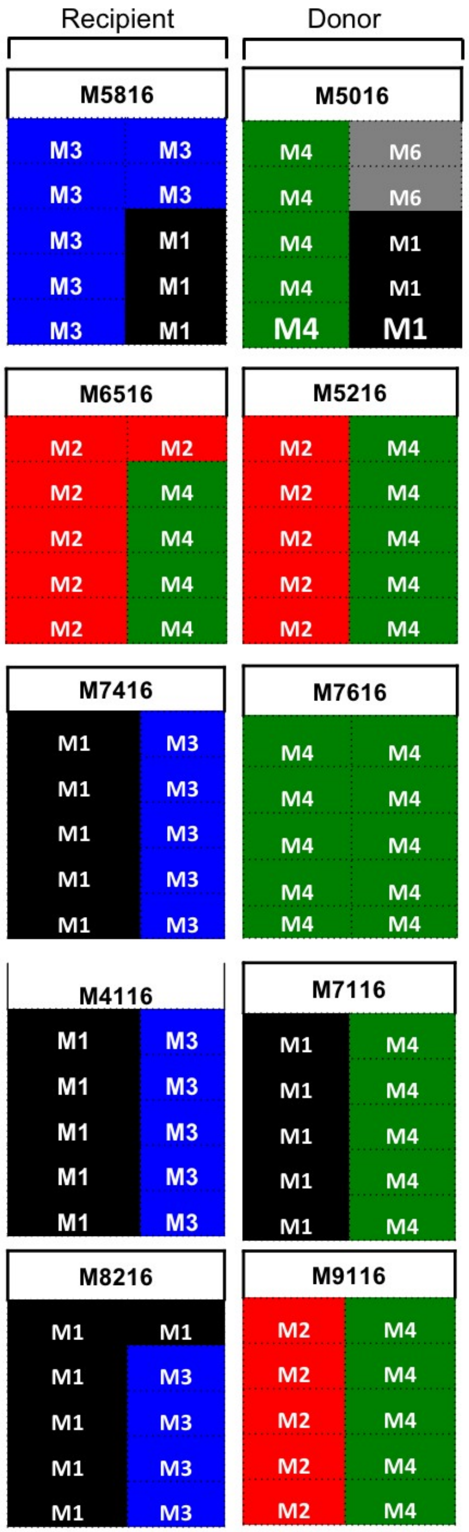

B

Organ	Recipient	Donor	MHC mismatch		DSA	Allograft Survival (Days after BMT)	Final Histology in Allo-organ	ISHLT Grading
			Class I	Class II				
Kidney*	M3312	M3212	4/4	6/6	-	449	TOLS, no rejection	-
Kidney*	M3215	M3615	4/4	6/6	-	108[1]	No rejection	-
Kidney*	M3515	M4813	4/4	6/6	+/-	540	TOLS, no rejection	-
Kidney*	M8014	M7914	4/4	6/6	-	728	TOLS, mild AMR, C4d pos	
Lung	M5816	M5016	4/4	3/6	-	41[2]	ACR, PTLD	A1 B1R, C4d negative
Lung	M6516	M5216	1/4	0/6	-	72[2]	Chronic interstitial pneumonitis, PTLD	A1 B1R, C4d negative
Lung	M7416	M7616	4/4	6/6	-	70	ACR	A4 B2R, AMR, C4d positive
Lung	M4116	M7116	2/4	3/6	-	42	ACR	A4, B2R, C4d negative
Lung	M8216	M9116	4/4	6/6	+	78[3]	AMR, ACR	A1 B1R, AMR, C4d positive

TOLS: Treg-rich organized lymphoid structures, ACR: Acute Cellular Rejection, AMR: Antibody-mediated Rejection, PTLD: Posttransplant lymphoproliferative disease [1]died due to anesthesia complication [2]euthanized for PTLD [3]after Organ Transplantation before BMT; * previously published (2)

Suppl. Fig. 2

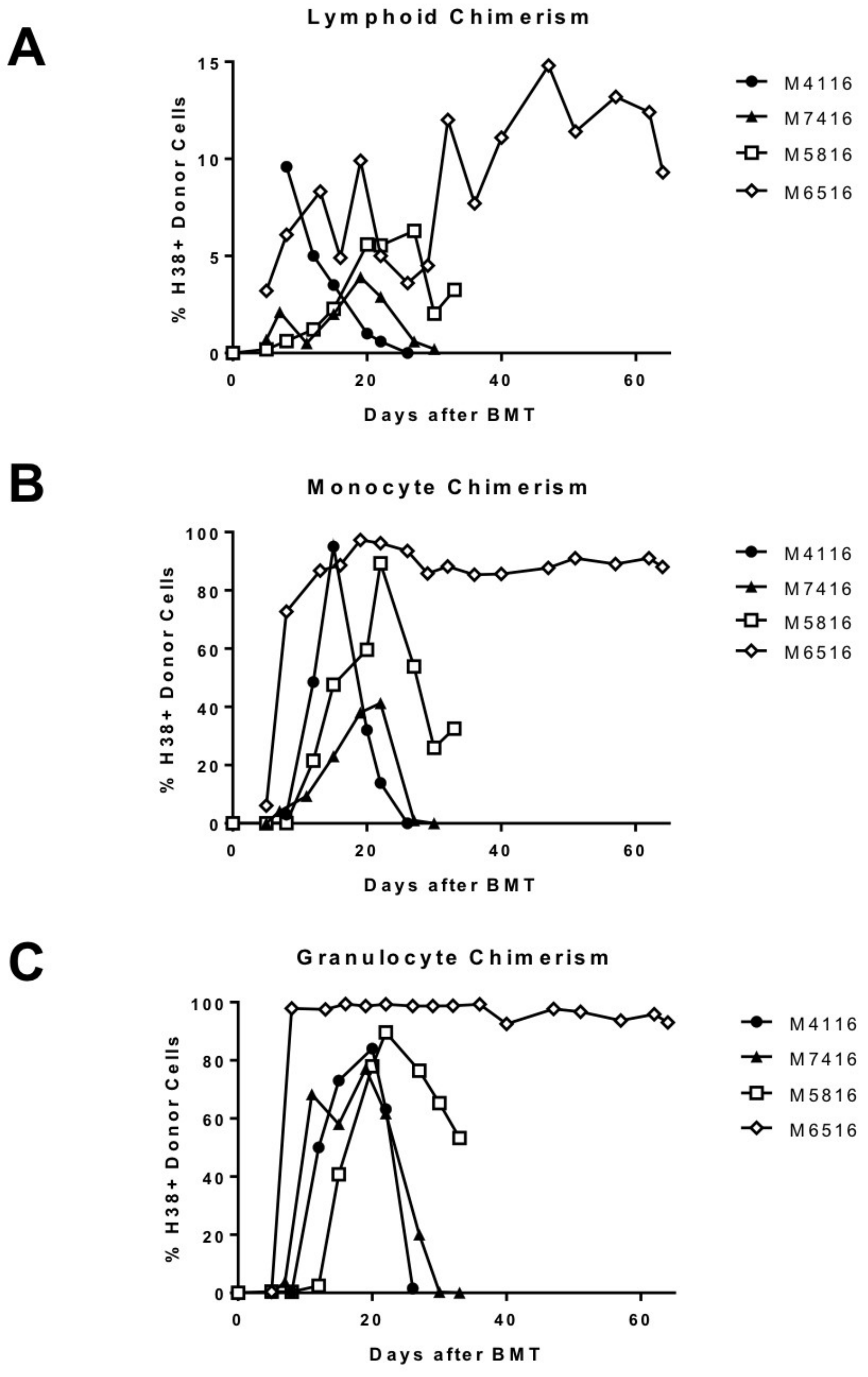
A
Lymphoid Chimerism
% H38+ Donor Cells
Days after BMT
M4116
M7416
M5816
M6516
B
Monocyte Chimerism
% H38+ Donor Cells
Days after BMT
M4116
M7416
M5816
M6516
C
Granulocyte Chimerism
% H38+ Donor Cells
Days after BMT
M4116
M7416
M5816
M6516

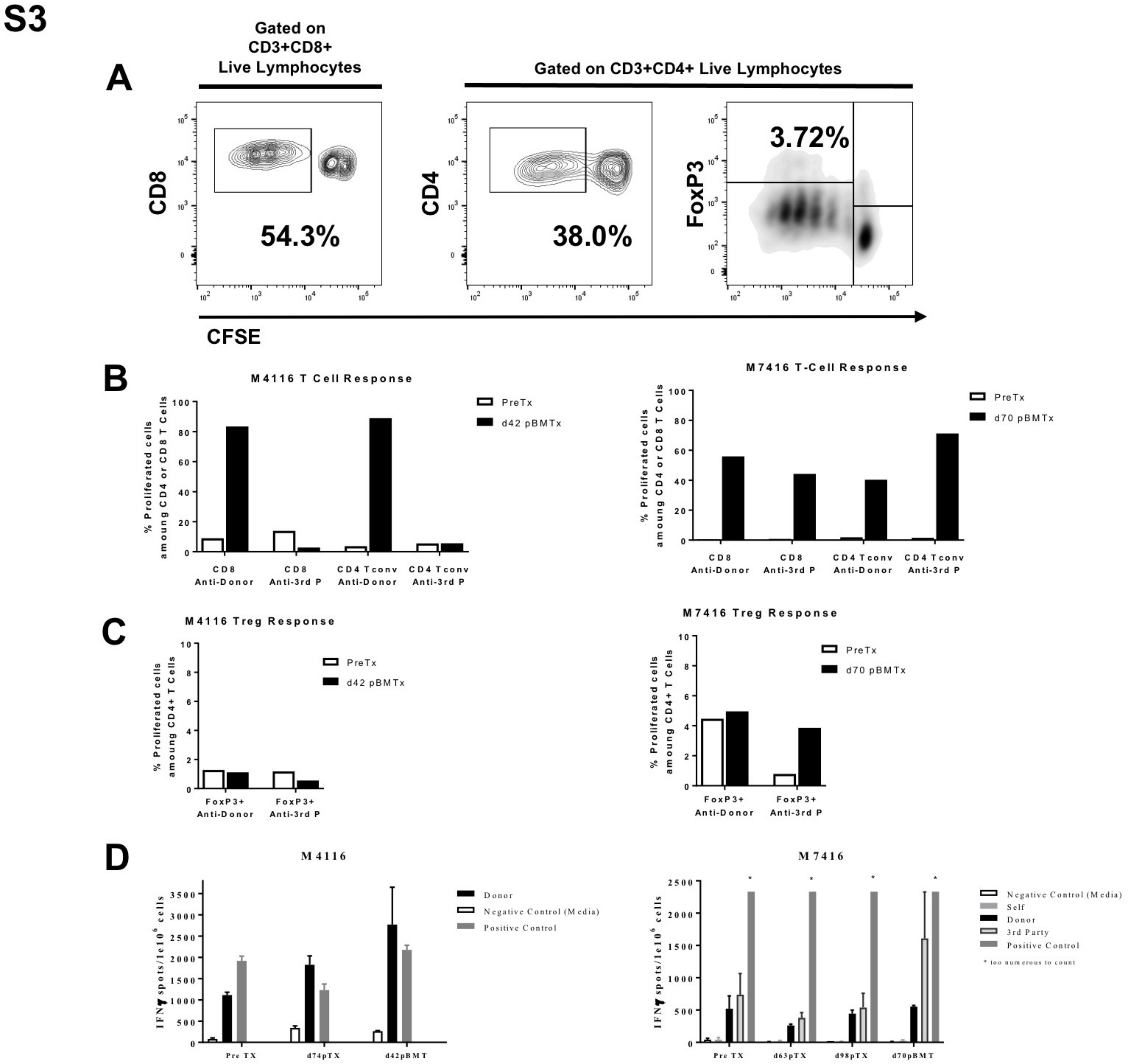

S3
A
Gated on CD3+CD8+ Live Lymphocytes
Gated on CD3+CD4+ Live Lymphocytes
CD8
54.3%
CD4
38.0%
FoxP3
3.72%
CFSE
B
M4116 T Cell Response
% Proliferated cells amoung CD4 or CD8 T Cells
PreTx
d42 pBMTx
CD8 Anti-Donor
CD8 Anti-3rd P
CD4 Tconv Anti-Donor
CD4 Tconv Anti-3rd P
M7416 T-Cell Response
PreTx
d70 pBMTx
C
M4116 Treg Response
% Proliferated cells amoung CD4+ T Cells
FoxP3+ Anti-Donor
FoxP3+ Anti-3rd P
M7416 Treg Response
D
M4116
Donor
Negative Control (Media)
Positive Control
Pre TX
d74pTX
d42pBMT
M7416
Self
3rd Party
* too numerous to count
Pre TX
d63pTX
d98pTX
d70pBMT
